# Mechanism of Fatty Acid Metabolism and Regulation by Lactate During Exercise in White Adipose and Skeletal Muscle Tissue: A Review

**DOI:** 10.1186/s40798-025-00862-5

**Published:** 2025-06-15

**Authors:** Shouzhen Huang, Ruonan Shangguan, Siyu Chen, Xiangdeng Lai, Haijun Han, Jingquan Sun

**Affiliations:** 1 Institute of Sports Science, Sichuan University, Chengdu, 610065 China; 2 College of Physical Education, Chengdu University, Chengdu, 610106 China; 3 Institute of Cardiology and Sports Medicine, German Sport University Cologne, 50933 Cologne, Germany; 4 School of Physical Education, Sichuan University, Chengdu, 610065 China

**Keywords:** Exercise, Lactate, Fatty acid, Adrenaline, Metabolism

## Abstract

Lactate plays a central role in controlling the utilization of energy substrates and the selection of metabolic pathways. This review aims to determine how lactate participates in energy supply and elaborate on how lactate is involved in the fat metabolism and regulation of white adipose and skeletal muscle tissues during exercise, thereby helping the human body achieve precise matching with different exercise intensities and a dynamic balance in energy supply.

Numerous studies have confirmed that lactate, through multiple pathways such as the GPR81 receptor and MCT1, regulates the cAMP/PKA signaling pathway, adrenaline concentration, and mitochondrial biogenesis and antioxidant function during exercise, participating in the fatty acid metabolism process of a single bout of exercise and exhibiting different effects in white adipose tissue and skeletal muscle, thereby effectively regulating lipid metabolism. This regulatory process is dependent on lactate concentration and exercise duration. Furthermore, lactate plays a crucial role in the restructuring of lipid metabolism induced by long-term exercise, particularly in promoting the browning of white adipose tissue and enhancing mitochondrial function. However, the bridging role of lactate in the transition of energy supply mechanisms and its deeper mechanisms in lipid metabolism regulation remain at the forefront of metabolic scientific research. In the future, there is an urgent need to delve into the regulatory network of lactate under different exercise intensities, reveal its potential applications in the treatment of metabolic diseases, provide a theoretical basis for the development of new treatment strategies, and promote the formulation of personalized exercise prescriptions to optimize metabolic health and disease management.

## Key Points

• Lactate regulates the cAMP/PKA signaling pathway, adrenaline concentration, and mitochondrial function through receptors such as GPR81 and transporters like MCT1, thereby affecting lipid metabolism in white adipose and skeletal muscle tissues during exercise.

• The regulatory effects of lactate on fatty acid metabolism are concentration and exercise duration dependent, with significant differences in its effects on white adipose and skeletal muscle tissues at different exercise intensities, highlighting the importance of lactate in energy metabolism and metabolic health.

• Lactate plays a crucial role in the restructuring of lipid metabolism induced by long-term exercise, particularly in promoting the browning of white adipose tissue and enhancing mitochondrial biogenesis and antioxidant function, which has implications for the prevention and treatment of obesity and metabolic syndrome.

## Background

During the exercise process, the human body must adjust its energy supply and metabolic mechanisms to meet the demands of different exercise intensities [1]. Adipose tissue, as a crucial energy reservoir in the human body, plays a central role in energy metabolism [2]. The fatty acids produced through its breakdown are not only a key source of energy for oxidative metabolism but also important participants in metabolic regulation, involving the control of various metabolic processes in the human body [3, 4]. As exercise intensity increases, the mechanisms of energy supply change accordingly, which in turn affects metabolic processes and the regulatory mechanisms of fatty acid metabolism. The maximum rate of fat oxidation in the human body is typically lower than an individual’s lactate threshold [5]. Due to individual differences, the lactate threshold can occur over a wide range of VO2max percentages [6]. Therefore, during moderate-intensity continuous training, if an individual has not reached their lactate threshold, then fatty acids are their primary energy source. Fatty acids are primarily provided by the breakdown of lipid droplets in white adipose tissue and skeletal muscle, with white adipose tissue playing a dominant role [7]. Research indicates that with the increase in exercise intensity, the overall proportion of fatty acid energy supply gradually decreases, and the contribution ratio of white adipose tissue and skeletal muscle tissue to energy supply also undergoes different changes. Currently, the specific connection between the proportion differences of these two tissues in fatty acid energy supply and energy regulation mechanisms is not yet fully clear.

Lactate, as the end product of glycolytic metabolism, also serves as an important physiological signaling molecule, with rapid developments in the field of signal transduction. The lactate shuttle theory explains that lactate can be transported both between cells and within cells. This transport allows lactate to serve as a bridge between glycolysis and the mitochondrial pathway. In recent years, research has found that lactate can not only act independently but also synergize with other signaling molecules, demonstrating complex functions of autocrine, paracrine, and endocrine [8]. Studies have shown that lactate can participate in the regulation of fatty acid metabolism and may be involved in the regulation of fatty acid metabolism in white adipose and skeletal muscle tissues [9]. Specifically, Many animal experiments have shown that lactate not only binds to the G protein-coupled receptor 81 (GPR81) to inhibit lipolysis in white adipose tissue [10], but also promotes lipolysis and the browning of white adipose tissue under certain conditions [11], and it has been found to promote the accumulation of intramuscular triglycerides (IMTG) in skeletal muscle through GPR81 [12, 13]. That is, the regulatory effect of lactate on lipid metabolism has a certain tissue specificity: in white adipose tissue (16mM) [14–16] and skeletal muscle tissue (5mM) [17–21], lactate has different regulatory effects on lipid metabolism, and even shows completely opposite functions at specific concentrations. Notably, exercise-induced elevation of lactate concentrations may provide physiological relevance to these mechanisms, though their causal interplay requires further validation in exercise models. These characteristics of lactate make it a substance that may have a profound impact on the field of energy metabolism [22]. Given the important role of lactate as a metabolic product of exercise on lipid metabolism, and its heterogeneity in the effects on lipid metabolism of white adipose and skeletal muscle tissues, it is receiving more and more attention from scholars.

This review article discusses the impact of lactate on fat metabolism during exercise, the mechanisms by which lactate regulates fat metabolism in white adipose and skeletal muscle tissues, and the functions of lactate in energy regulation during acute exercise and in promoting fat breakdown with long-term exercise. It elucidates how lactate is involved in the regulation of fat metabolism in white adipose and skeletal muscle tissues during exercise, which contributes to a deeper understanding of the role of lactate in regulating energy metabolic substrates and its potential implications for obesity and related chronic diseases. The databases Web of Science, Google Scholar, PubMed, and Science Direct were used to search the related literatures. A Search of the literature using “physical activity/exercise/sport/metabolism” + “adipose/fatty acid/lactate/skeletal muscle” as keywords was conducted. The first literature search was conducted in October 2023, during which 189 relevant articles were identified. The second literature search was conducted in August 2024, and it identified 44 additional articles. The final reference list for this article has been determined, after selecting newer articles with similar content, retaining classic literature, excluding duplicates, and considering the modification suggestions.

## Overview of Energy Supply by Energy Substrate During an Aerobic Exercise

During a single bout of exercise, energy supply primarily relies on three major energy systems: the phosphagen system, the glycolytic system, and the aerobic oxidative system. These systems play their respective roles depending on the intensity and duration of the exercise. The oxidative system takes a dominant role after more than 2 min of exercise. In this oxidative metabolism process, the accumulation of lactate becomes an important regulatory factor. The continuous accumulation of lactate during exercise can inhibit further glycolysis, forcing the body to rely more on the aerobic oxidative system [24]. At the same time, the body clears lactate through various mechanisms, including gluconeogenesis and oxidation as an energy source. Additionally, animal experiments have shown that lactate helps regulate pH by cotransporting with H + across MCTs [8, 9].

Human studies have shown that during oxidative metabolism processes, the main sources of energy include muscle glycogen (MG), intramuscular triglycerides (IMTG), plasma free fatty acids (Plasma FFA), and plasma glucose (Plasma G). As the intensity of exercise increases, the energy supply ratio of carbohydrates to fats also changes. For instance, at an exercise intensity of 25% VO2 max, the relative contributions are: Plasma FFA > IMTG > Plasma G, with MG not typically included at this intensity. At 65% VO2 max, the contributions are: MG > Plasma FFA > IMTG > Plasma G. And at 85% VO2 max, the contributions are: MG > Plasma FFA > Plasma G > IMTG. These contributions are expressed in cal/kg/min [7, 25]. Therefore, exercise at moderate to low intensities is more conducive to promoting the breakdown of white adipose tissue. However, the mechanism behind this shift in energy supply is not yet fully understood. This article will attempt to explore the mechanism of this change in energy supply ratio from the perspective of lactate, drawing on insights from animal experiments, in hopes of gaining a deeper understanding of this phenomenon.

## Changes of Fatty Acid Source and Lactate Concentration During a Single Bout of Aerobic Exercise

**Fig. 1:**
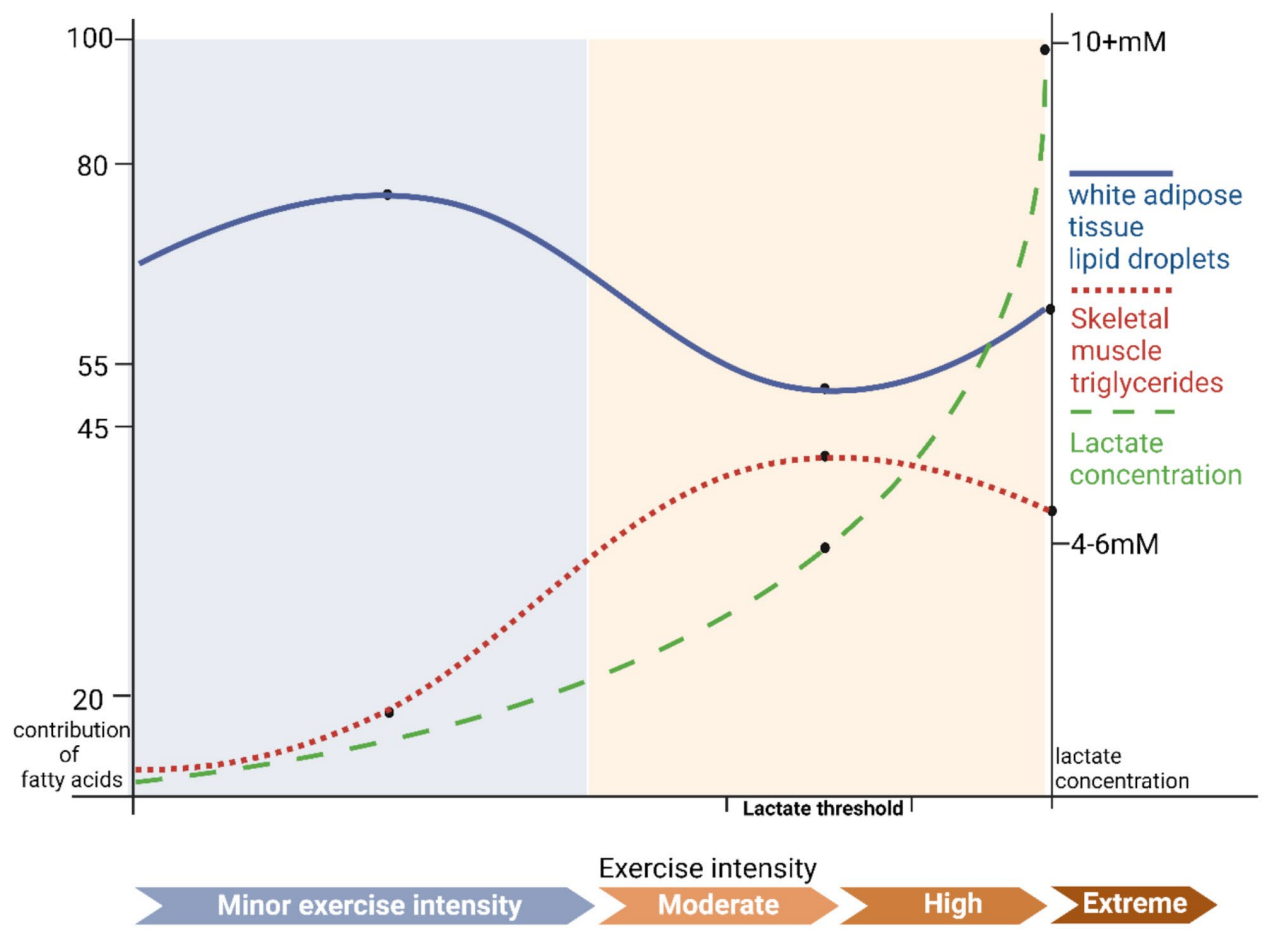
Fatty acid contribution from white adipose tissue and skeletal muscle tissue

Past human experimental data indicate that the adjustment of fatty acid contributions from white adipose and skeletal muscle tissues may be related to the continuous production of lactate [14, 15, 26]. Specifically, when blood lactate concentration is around 2 mM(Aerobic Threshold), fatty acids are mainly derived from the breakdown of lipid droplets in white adipose tissue. As blood lactate concentration increases to approximately 5 mM(Anaerobic Threshold), the contribution of white adipose tissue to fatty acids gradually decreases. However, when blood lactate concentration exceeds approximately 5 mM, the contribution ratio of white adipose tissue to fatty acid energy sources remains higher than that of IMTG, as plasma free fatty acids from white adipose tissue are consistently more abundant. At lower blood lactate concentrations, the proportion of fatty acids provided by the breakdown of IMTG increases, reaching its peak at a blood lactate concentration of approximately 5 mM, and then decreases with further increases in lactate concentration [7] (see Fig. 1 for details). It is worth noting that at blood lactate concentrations above approximately 16 mM, fat oxidation is almost halted [26]. Therefore, the contribution of fatty acids at such high lactate concentrations is not depicted in the figure (the rate of lactate production and the threshold can vary among individuals due to differences in exercise capacity, and the trend of lactate concentration changes shown in the figure is based on the general situation of the general population).

This figure is adapted from Fig. 3 in reference [7], which itself is adapted from the original manuscript [25]. The lactate concentrations shown in the figure are referenced from [7, 25, 27, 28]. The blue shade in the background represents rest and low-intensity exercise, while the yellow shade indicates moderate and higher intensities. Created with BioRender.com.

## Different Effects of Lactate in White Adipose and Skeletal Muscle Tissue

Previous studies have confirmed that lactate can inhibit lipolysis in white adipose tissue through GPR81 and limit the uptake of fatty acids by muscle mitochondria by affecting malonyl-CoA and Carnitine Palmitoyl Transferase 1 (CPT1) [10, 29]. This mechanism promotes the muscle’s reliance on glucose as an energy source, thereby finely regulating fatty acid metabolism and energy supply. However, previous animal experiments studies have shown that intramuscularly injected lactate can promote lipolysis and browning in white adipose tissue [11]. These effects were observed in mice, indicating that lactate can induce lipolysis and browning when administered intramuscularly. This indicates that the role of lactate in white adipose tissue is bidirectional, as it can both inhibit lipolysis and promote lipolysis and tissue browning under specific conditions. In addition, animal research has found that lactate promotes the accumulation of IMTG in skeletal muscle through GPR81 [12, 13]. With exogenous lactate injections, a significant increase in the expression of proteins related to fat synthesis and mitochondrial biogenesis in mouse skeletal muscle has been observed, while inhibiting fat breakdown in the gastrocnemius muscle [30, 31]. These results suggest that in skeletal muscle tissue, lactate can inhibit fat breakdown and promote synthesis, accompanied by enhanced mitochondrial biogenesis. In white adipose tissue, lactate has a completely different effect on lipid metabolism, which may be due to lactate’s ability to increase the expression of Beta-3 adrenergic receptor (β3-AR) in white adipose tissue [11], and/or the activation of the sympathetic nervous system. Exercise-induced fluctuations in blood glucose may lead to increased activity of adrenergic signals in adipose tissue, while activity in skeletal muscle may remain unaffected [18, 20, 32].

The specific mechanisms by which lactate exerts different effects on fat metabolism and regulation in white adipose and skeletal muscle tissues may be related to a variety of factors, including the lactate concentrations used by researchers, the observation/sampling time, and differences in the subjects of study (such as cellular/animal models, etc.). Currently, these mechanisms are not fully understood and require further research for clarification.

## Lactate Is Involved in the Regulation of Fatty Acid Metabolism and its Possible Mechanism

Lactate is produced from pyruvate through the action of lactate dehydrogenase and is often considered an indicator of the shift in the energy supply ratio between carbohydrates and fats during exercise. As a type of exercise metabolite, lactate can serve as an energy substrate in the circulatory system or participate in the metabolic processes of organs and cells through autocrine, endocrine, and paracrine pathways [8]. Lactate can be involved in the regulation of fatty acid metabolism through various molecular pathways such as ion permeation [33], the family of G protein-coupled receptors [34], and the family of monocarboxylate transporters (MCT) [35–39]. Current research indicates that the intake of exogenous lactate, either orally or through injection, can induce a reshaping of fat metabolic processes. Lactate itself can also influence the metabolic progression of adipose tissue by modulating various factors related to lipogenesis, lipolysis, and thermogenesis associated with browning, thereby improving obesity and related metabolic disorders to a certain extent [40]. This suggests that lactate plays a significant role in fatty acid metabolism (as shown in Table 1).

**Table 1:** Lactate-mediated regulation of fatty acid metabolism

Author	Research target	Experimental subject	Research results
Liu. C, Wu. J, Zhu. J. et al. [10]	GPR81	GPR81 gene knockout mice	Lactate suppresses lipolysis in adipose tissue through a direct activation of GPR81.
K. Ahmed, S. Tunaru, C. et al. [16]	GPR81	GPR81 gene knockout mice	Lactate inhibits the cAMP/PKA pathway and regulates fat metabolism in a hormone-like manner.
S. Chen, L. Zhou, J. Sun, Y. Qu and M. Chen [31]	cAMP/PKA	Mice	Lactate inhibits the cAMP/PKA pathway and promotes triglyceride accumulation in skeletal muscle, as well as stimulates mitochondrial biogenesis.
Kitaoka, Y. Takeda, K. Tamura, Y. et al. [41]	PGC-1α and UCP3	Mice	Increased expression of Ppargc1a, Pdk4, and Ucp3 was confirmed using real-time quantitative polymerase chain reaction.
Carrière, A. Jeanson, Y. Berger-Müller, S. et al. [42]	UCP1	Mice	Lactate-induced browning also occurs in human cells and in vivo. Lactate controls Ucp1 expression independently of hypoxia-inducible factor-1α and PPARα pathways but requires active PPARγ signaling.
N. Esaki, T. Matsui and T. Tsuda [43]	UCP1	Mice	Lactate promotes the expression of UCP1 and induces adipose tissue browning by increasing the levels of reactive oxygen species (ROS) and the NADH/NAD+.
P. Hojman, C. Brolin, N. et al. [44]	IL-6	Healthy young males/Mice	During exercise, muscle releases IL-6, and this release is stimulated by lactate-dependent protease activity.
Y. Jeanson, F. Ribas, A. Galinier, et al. [45]	FGF21	Mice	Lactate induces the expression of FGF21 in adipocytes through the activation of the p38-MAPK pathway.
Latham. T, Mackay. L, Sproul. D. et al. [46]	Histone acetylation	HCT116 cells	Primary effect of HDAC inhibition by endogenous short-chain fatty acids like lactate is to promote gene expression at genes associated with HDAC proteins.
Y. J. An, S. Jo, J. et al. [47]	Histone acetylation	Purified cell nucleus	Lactate regulates histone acetylation through nuclear LDH metabolism.
Takahashi, K. Kitaoka, Y. Matsunaga. et al. [48]	Mitochondrial enzyme activity	Mice	Lactate administration increased mitochondrial enzyme activity (citrate synthase, 3-hydroxyacyl CoA dehydrogenase, and cytochrome c oxidase) in the plantaris muscle.
Takahashi, K. Tamura, Y. Kitaoka, Y. et al. [49]	Mitochondrial respiratory function	Mice	Lactate administration significantly enhanced pyruvate + malate- and glutamate + malate-induced (complex I-driven) state 3 (maximal/ATP synthesis-coupled) respiration.
X. Cai, C. P. Ng, O. et al. [50]	Mitochondrial electron transport chain	HepG2 cell	Lactate activates the mitochondrial electron transport chain and oxidative phosphorylation.

## The Role and Possible Mechanism of Lactate in Regulating Fatty Acid Metabolism in Adipose Tissue

**Fig. 2:**
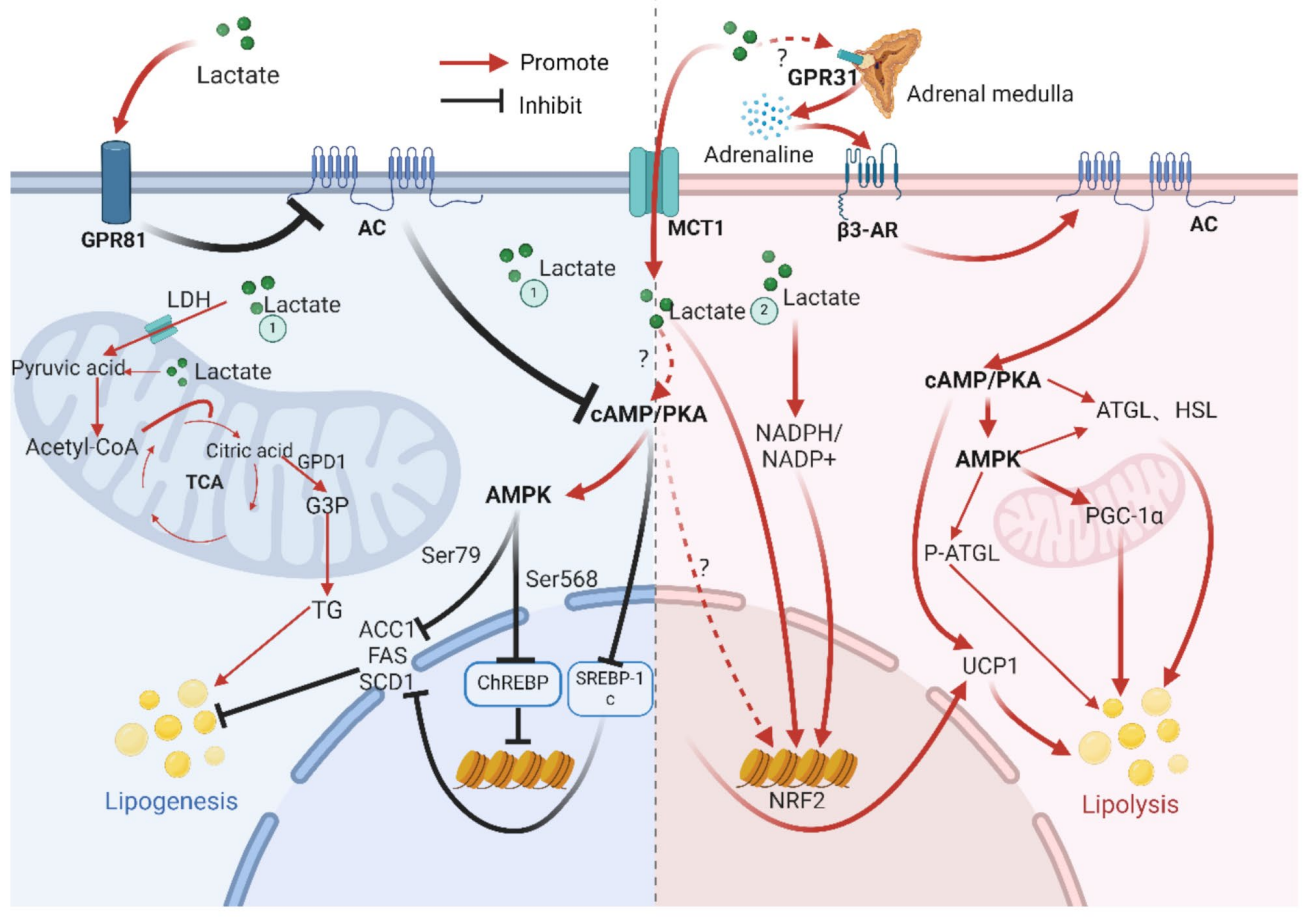
Mechanism of lactate regulation of fatty acid metabolism in adipose tissue

Many animal experiments studies suggest that in adipocytes, the direct function of lactate is to bind to GPR81, leading to the inhibition of adenylate cyclase, a decrease in cAMP production, and thus the inhibition of lipolysis [10, 16, 51]. There is also research indicating that lactate can promote the breakdown and browning of adipocytes. Therefore, this section mainly elaborates on the adipose tissue-specific regulation of fatty acid metabolism by lactate and its potential mechanisms (see Fig. 2 for details).

The blue sections indicate fatty acid synthesis, and the red sections indicate fatty acid breakdown. GPR81 (G Protein-Coupled Receptor 81), AC (Adenylyl Cyclase), MCT1 (Monocarboxylate Transporter 1), β3-AR (Beta-3 Adrenergic Receptor), GPR31 (G Protein-Coupled Receptor 31), LDH (Lactate Dehydrogenase), Acetyl-CoA (Acetyl Coenzyme A), TCA (Tricarboxylic Acid Cycle), GPD1 (Glycerol-3-Phosphate Dehydrogenase 1), G3P (Glyceraldehyde-3-Phosphate), TG (Triglyceride), cAMP/PKA (Cyclic AMP/Protein Kinase A), AMPK (AMP-Activated Protein Kinase), ACC1 (Acetyl-CoA Carboxylase 1), FAS (Fatty Acid Synthase), SCD1 (Stearoyl-CoA Desaturase 1), ChREBP (Carbohydrate-Responsive Element-Binding Protein), SREBP-1c (Sterol Regulatory Element-Binding Protein 1c), NADPH (Nicotinamide Adenine Dinucleotide Phosphate), NRF2 (Nuclear Factor Erythroid 2-Related Factor 2), ATGL (Adipose Triglyceride Lipase), HSL (Hormone-Sensitive Lipase), PGC-1α (Peroxisome Proliferator-Activated Receptor Gamma Coactivator 1 Alpha), UCP1 (Uncoupling Protein 1). Created with BioRender.com.

In white adipose tissue, regarding fatty acid synthesis, lactate not only serves as an energy substrate for the TCA cycle [52], but also enters the mitochondrial matrix, enhancing the activity of the Electron Transfer Chain (ETC) [50]. This increase in the NADPH/NADP + ratio boosts the activity of glycerol-3-phosphate dehydrogenase GPD1, thereby increasing the intracellular content of G3P, which is favorable for TG synthesis [53]. However, in white adipose tissue, lactate also exhibits an inhibitory effect on fat synthesis. Both acute and chronic injections of 0.25 M lactate (dose of 0.64 mL/kg) into the gastrocnemius muscle activate the cAMP/PKA signaling pathway in inguinal white adipose tissue, thereby inhibiting the fatty acid synthesis process. In white adipose tissue, the activation of the cAMP/PKA signaling pathway directly inhibits the synthesis of complex fatty acids such as malonyl CoA and palmitic acid by inhibiting the activity of ACC1, FAS, SREBP-1c, and SCD1 [54–56]. At the same time, it inhibits the DNA binding activity of ChREBP [57, 58] and reduces the content of PPARγ, which is the main transcription factor for adipogenesis [59]. Lactate promotes fatty acid synthesis through metabolic pathways, while inhibiting fatty acid synthesis through receptor pathways.

In terms of fatty acid breakdown, lactate has been found to increase the expression of β3-AR in white adipose tissue [11], and may promote the secretion of adrenaline through the GPR31 receptor(or the activation of the sympathetic nervous system to promote.) [60, 61]. Adrenaline activates the cAMP-PKA signaling pathway, phosphorylates ATGL and HSL, and stimulates fatty acid breakdown [62]. In white adipose tissue, the activation of the cAMP-PKA signaling pathway also leads to the activation of the AMPK pathway, which helps to promote the oxidative breakdown of fat and inhibits its synthetic process. AMPK phosphorylates acetyl-CoA carboxylase, reducing the generation of malonyl-CoA, thereby relieving the inhibitory effect on CPT1 and promoting the breakdown of FFA [63]; at the same time, in white adipose tissue, AMPK also increases the phosphorylation activity of ATGL, overall promoting fat breakdown; the activation of AMPK also promotes the expression of PGC-1α, which is a key regulatory molecule for mitochondrial biogenesis [64, 65], and can accelerate the oxidative breakdown of fat. Lactate can also increase oxidative phosphorylation through metabolic pathways, raise the NADPH/NADP + ratio, thereby inducing the upregulation of UCP1-related genes, and promoting the browning of adipose tissue [66].

## The Effect and Mechanism of Lactate on Fatty Acid Metabolism in Muscle Tissue

**Fig. 3:**
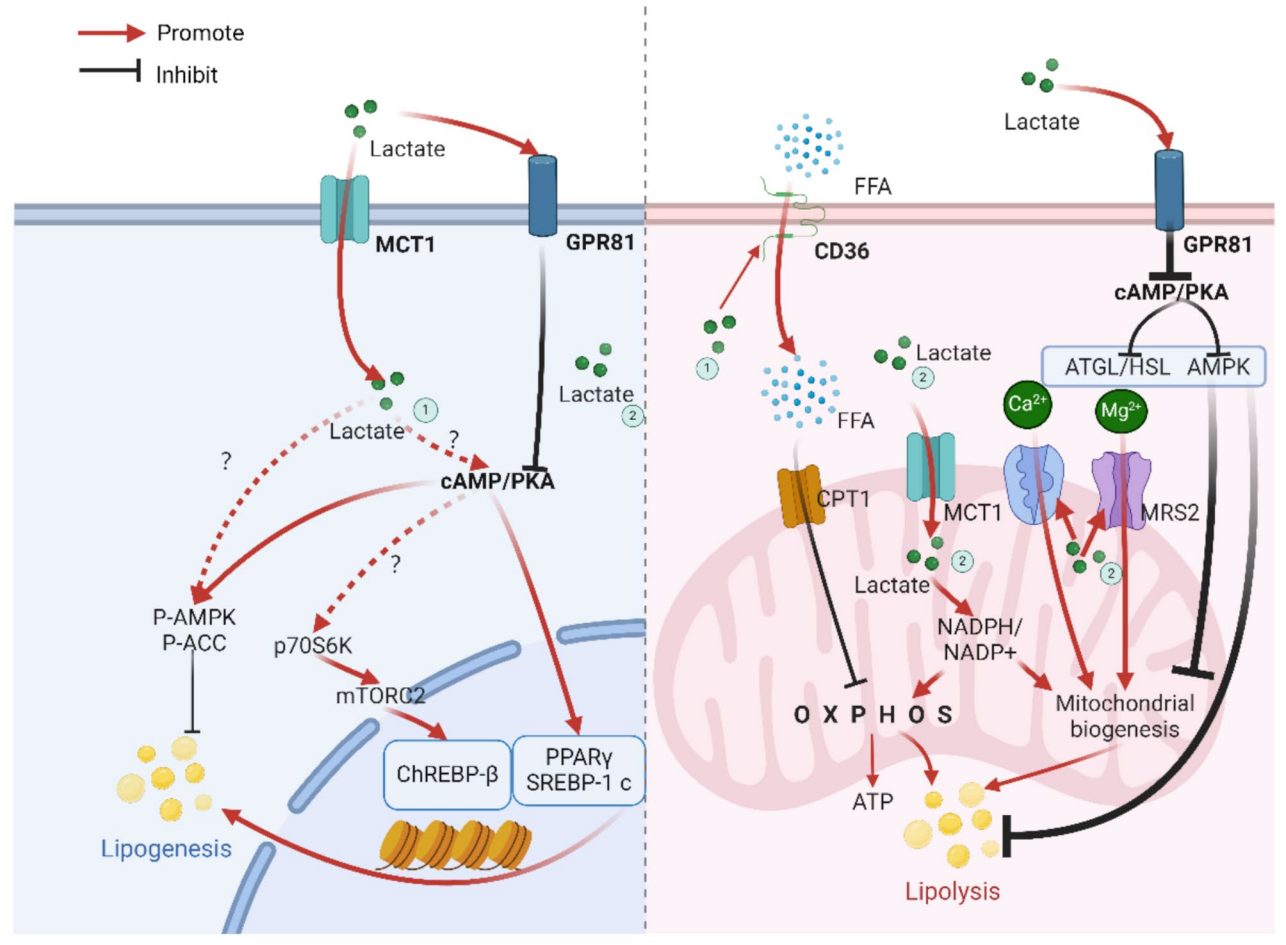
Mechanism of lactate regulation of fatty acid metabolism in muscle tissue

In mice that received a single or chronic injection of lactate (with a muscle lactate concentration of approximately 0.25 M) into the gastrocnemius muscle, a significant decrease was observed in the levels of AMPK, P-AMPK, P-HSL, CPT-1B, and TGF-β2 within the muscle tissue, while the markers of mitochondrial biogenesis and antioxidant function, PGC-1α and citrate synthase, increased [31, 41]. This suggests that fatty acid breakdown is inhibited, and mitochondrial function is enhanced. When C2C12 myotubes were treated with 16 mM lactate for 0.5 to 4.5 h, it was found that lactate could bind to GPR81, inhibit the cAMP signaling pathway, induce triglyceride (TG) accumulation, inhibit fat breakdown, and participate in the extracellular polymeric substance-induced TG accumulation and enhanced mitochondrial biogenesis in skeletal muscle cells. However, some muscle cell studies have also found that lactate, as a metabolic regulator, activates AMPK [67], thereby inhibiting fat synthesis. The contradictory phenomena mentioned above may be due to different concentrations and durations of lactate treatment, but they may also be related to the effects of Mg2 + and Ca2 + in animal experiments (see Fig. 3, as mentioned earlier, the role of adrenaline in skeletal muscle fatty acid metabolism is relatively stable, hence not presented in the figure [32]).

The blue sections indicate fatty acid synthesis, and the red sections indicate fatty acid breakdown. MCT1 (Monocarboxylate Transporter 1), GPR81 (G Protein-Coupled Receptor 81), cAMP/PKA (Cyclic AMP/Protein Kinase A), AMPK (AMP-Activated Protein Kinase), ACC (Acetyl-CoA Carboxylase), p70S6K (p70 Ribosomal S6 Kinase), ChREBP-β (Carbohydrate-Responsive Element-Binding Protein-β), PPARγ (Peroxisome Proliferator-Activated Receptor Gamma), SREBP-1c (Sterol Regulatory Element-Binding Protein 1c), FFA (Free Fatty Acid), CD36 (Cluster of Differentiation 36), CPT1 (Carnitine Palmitoyltransferase 1), MRS2 (Mitochondrial RNA Synthetase 2), ATGL (Adipose Triglyceride Lipase), HSL (Hormone-Sensitive Lipase), NADPH (Nicotinamide Adenine Dinucleotide Phosphate), OXPHOS (Oxidative Phosphorylation), ATP (Adenosine Triphosphate). The “?” in the figure represents uncertain mechanisms. Created with BioRender.com.

In the context of fat synthesis in skeletal muscle tissue, mice with chronic intra-muscular injections of lactate (blood lactate at 5mM) have been observed to have significant increases in PPARγ and SREBP-1c in the gastrocnemius muscle, promoting de novo fatty acid synthesis [30]. This may be related to GPR81 inhibiting the cAMP/PKA signaling pathway, which enhances the activity of enzymes related to fatty acid synthesis [12, 13]. Additionally, research has found that lactate can activate mTORC2 factor by stimulating the phosphorylation of p70S6K, thereby affecting the expression of ChREBP-β and promoting de novo fatty acid synthesis [68, 69].

Regarding fat breakdown in skeletal muscle tissue, lactate has been shown to reduce the activity of acetyl-CoA and CPT-1, inhibiting the uptake of free fatty acids (FFA) [8], indicating an inhibitory effect on fatty acid breakdown. Moreover, lactate, upon binding to the GPR81 receptor, suppresses the cAMP/PKA signaling pathway, thereby inhibiting the activity of fat breakdown-related enzymes such as ATGL and HSL, as well as the activity of mitochondrial-related enzymes like CREB and PGC-1α, and mitochondrial biogenesis [30, 31]. However, lactate also has the role of promoting the oxidation of fatty acids in muscle tissue for energy supply. Studies have found that after oral administration of lactate at 2 g/kg to mice for 60 min, the expression of CD36 in the muscle increased [70], enhancing the muscle’s ability to take up fatty acids and promoting the oxidation of fatty acids for energy supply. In addition to affecting the expression of fat breakdown-related proteins, lactate can also influence metabolic processes by affecting ion concentrations. Research has indicated that lactate can directly enter the mitochondrial matrix through MCT1, increasing the flux of the ETC, and stimulating the Mg2 + ion channel MRS2, allowing Mg2 + ions to enter the mitochondria and thus promoting the rate of oxidative phosphorylation, which gradually increases to a maximum value at 2-5mM concentrations. However, when lactate concentrations exceed 5mM, the excessive Mg2 + entering the mitochondria can inhibit the rate of oxidative phosphorylation [21]. Treatment with lactate can also increase mitochondrial Ca2 + levels, promoting mitochondrial biogenesis [21, 71]. Therefore, lactate may inhibit fatty acid breakdown through receptor pathways, while promoting fatty acid oxidation through metabolic pathways, thus affecting fatty acid breakdown metabolism.

## Lactate may be Involved in Regulating the Energy Supply of White Adipose and Skeletal Muscle Tissue During One-time Exercise

**Fig. 4:**
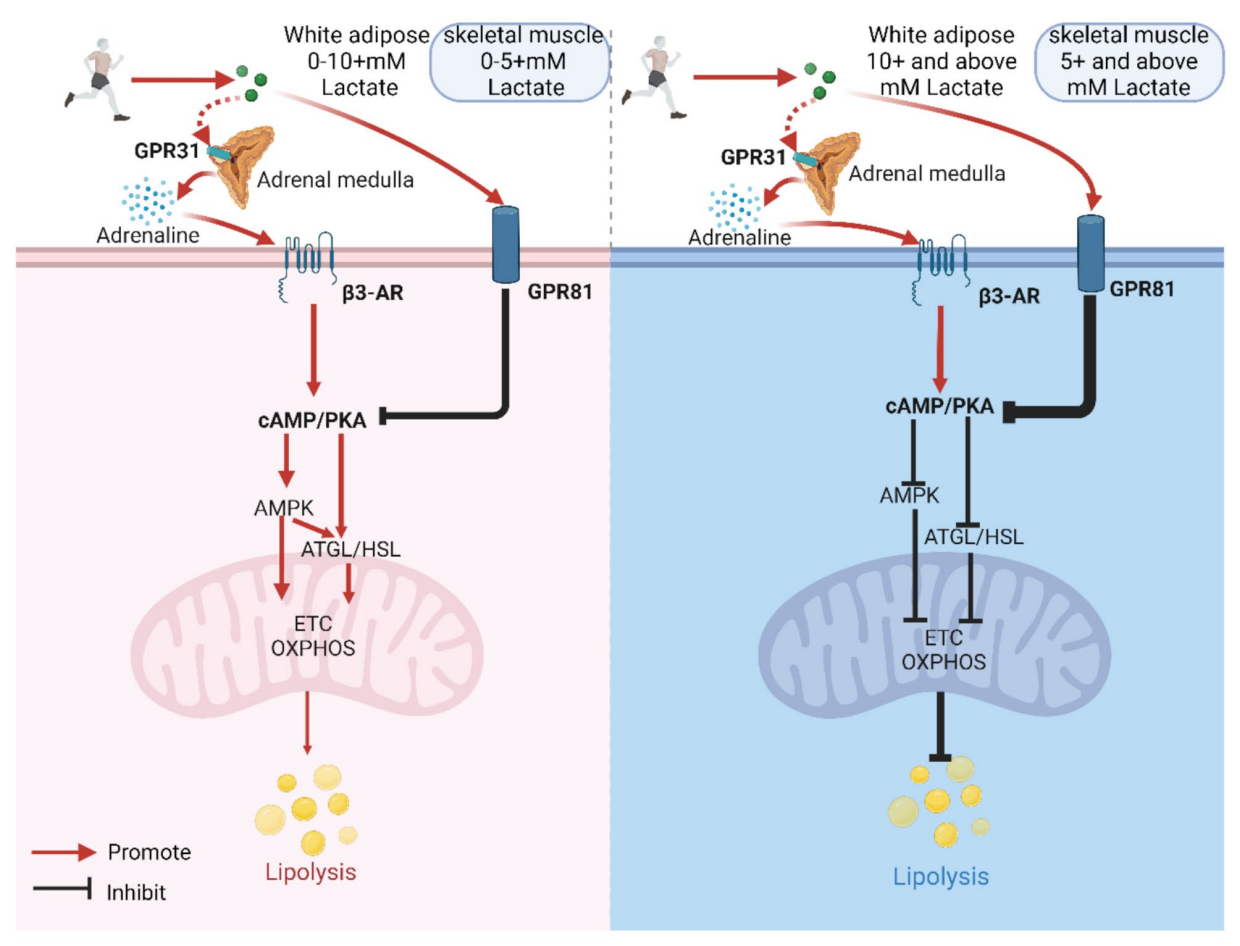
Possible mechanisms by which different concentrations of lactate regulate fatty acid

In a single bout of exercise, the amount of lactate produced at different intensities was not previously thought to have any significant differences. However, recent research suggests that this indispensable metabolic product of exercise is not merely a side effect of energy conversion but also a complex regulator that subtly builds a bridge between fat synthesis and breakdown. In white adipose tissue and skeletal muscle, lactate exhibits its unique dual identity, differentially regulating energy storage and release. This process is finely regulated by various factors such as lactate concentration, hormone levels, and receptor numbers, with the cAMP/PKA signaling pathway playing a central role (see Fig. 4 for details).

Added caveats regarding GPR31 involvement: Original references 32–33 focused on intestinal cells, and no direct evidence exists for GPR31 mediating lactate-adrenaline interactions in adrenal medulla. In past human studies, Fattor et al. [72] demonstrated that exogenous lactate infusion can inhibit the catecholamine response during exercise, and Veneman et al. [73] reported lower concentrations of epinephrine and norepinephrine during supraphysiological lactate infusion(> 4 m.

M). However, recent studies suggest that endogenous lactate accumulation under stress (e.g., exercise) may exhibit complex interactions with catecholamine secretion, potentially involving both inhibitory and stimulatory pathways depending on concentration thresholds and physiological context. For instance, while Fattor et al. observed catecholamine suppression at blood lactate concentrations of ~ 4 mM during exercise, endogenous lactate accumulation (e.g., 0–10 mM) in other contexts has been linked to indirect activation of sympathetic pathways via central or peripheral chemoreceptors [74–80], though direct evidence linking lactate to adrenaline secretion during exercise remains limited. Therefore, the biphasic effects of lactate on catecholamines may depend on its source and temporal dynamics: Acute exogenous lactate infusion (> 4 mM) activates peripheral inhibitory receptors (e.g., GPR81), suppressing catecholamines. Gradual endogenous accumulation (0–10 mM) during exercise might enhance sympathetic activity via central pathways (e.g., carotid body or hypothalamic activation), though this requires direct experimental validation. Critically, Fattor et al.‘s findings demonstrate that even within the 0–10 mM range, lactate can suppress catecholamines under specific conditions (e.g., rapid infusion during exercise), highlighting the need for mechanistic studies disentangling lactate’s source and kinetics. The discussion below is based on the conjectured scope of the relevant studies. In the background, red indicates overall promotion, and blue indicates overall inhibition. GPR31 (G Protein-Coupled Receptor 31), β3-AR (Beta-3 Adrenergic Receptor), GPR81 (G Protein-Coupled Receptor 81), cAMP/PKA (Cyclic AMP/Protein Kinase A), AMPK (AMP-Activated Protein Kinase), ATGL (Adipose Triglyceride Lipase), HSL (Hormone-Sensitive Lipase), ETC (Electron Transport Chain), OXPHOS (Oxidative Phosphorylation). Created with BioRender.com.

We hereby declare that the proposed thresholds are tentative and context-dependent, influenced by factors such as exercise duration, tissue-specific receptor expression (e.g., differential density of GPR81 in adipose tissue versus skeletal muscle, as well as the distinct expression of adrenergic signaling in adipose and skeletal muscle tissues), and the temporal dynamics of adrenaline release and clearance. In white adipose tissue, when the blood lactate concentration is around 0–10 mM [14–16], lactate may act as a signaling molecule of the nervous system (via hypothalamic-sympathetic pathways) [60, 61, 77], stimulating the secretion of catecholamines such as adrenaline, and increasing the expression of β3-AR in adipose tissue [11], promoting the breakdown of adipose tissue. It is well known that exercise induces an increase in serum adrenaline and lactate content [81]. A 2010 report in Cell Metabolism indicated that GPR81 does not play a key role in regulating fat breakdown during moderate to high-intensity exercise (blood lactate concentration of 4–10 mM) [16]. Therefore, the exercise intensity-dependent role of lactate in regulating fatty acid metabolism in white adipose tissue during a single bout of exercise may be as follows: At blood lactate concentrations < 5 mM, adrenaline-driven lipolysis via β3-AR may dominate over lactate-GPR81 signaling, potentially due to exercise-induced sympathetic activation rather than direct lactate action; when the blood lactate concentration is around 5–10 mM, lactate might further enhance the content and function of adrenaline, activating the cAMP/PKA signaling pathway through β3-AR more than lactate binding to GPR81 inhibits it, overall promoting the breakdown of fatty acids. when the blood lactate concentration is greater than 10 mM, lactate binds extensively with GPR81, leading to the inhibition of adenylate cyclase, thereby inhibiting the cAMP/PKA signaling pathway and the breakdown of fat [10, 16, 51], the effect of lactate-mediated adrenaline to promote the cAMP/PKA signaling pathway is reversed, and overall it inhibits the breakdown of fatty acids.

**Fig. 5:**
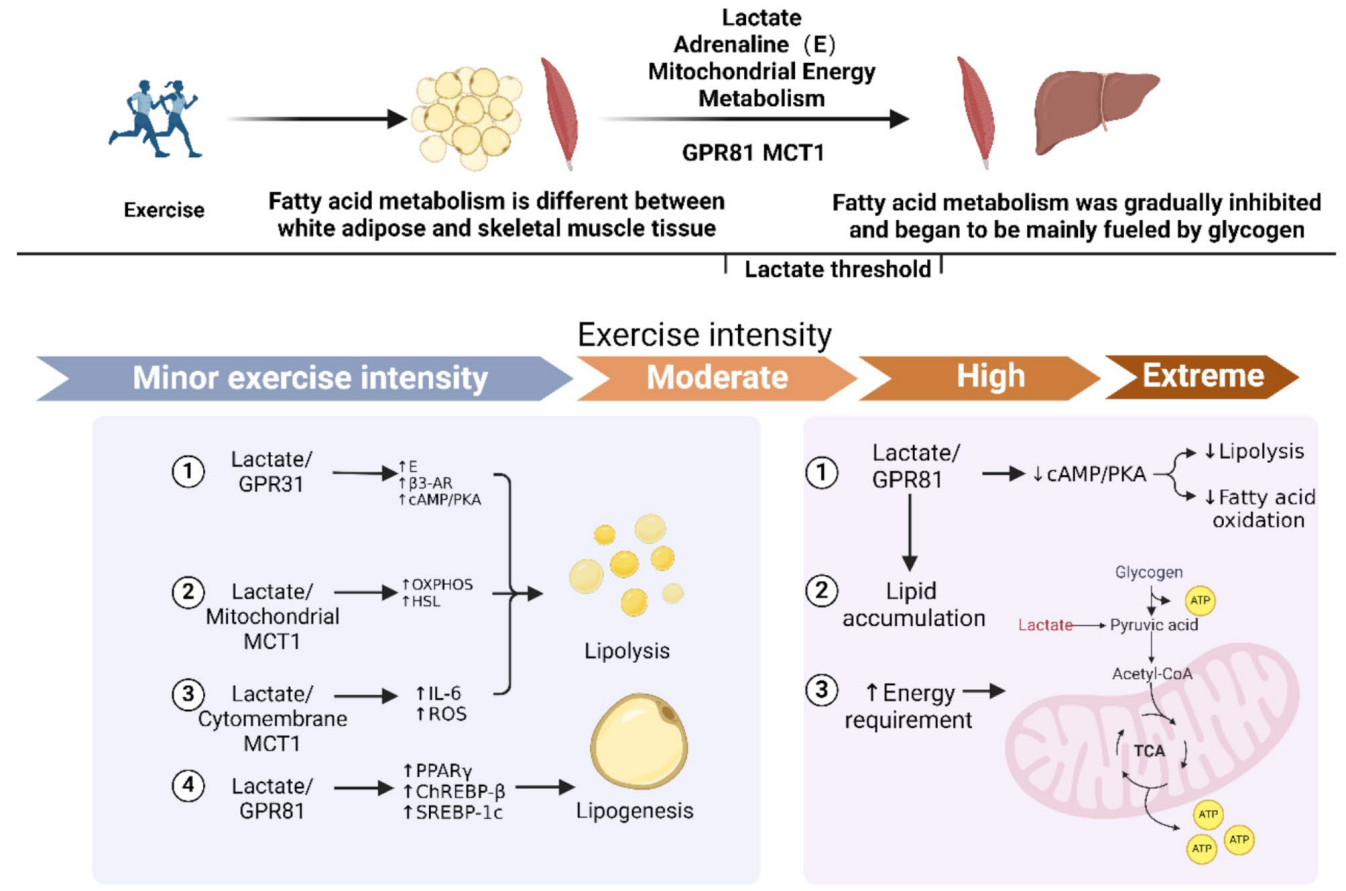
Lactate is involved in the regulation of energy metabolism during exercise

During exercise, serum adrenaline concentrations increase continuously. When the blood lactate concentration reaches around 5 mM [15], this coincides with reduced skeletal muscle uptake of plasma FFA [17], and maximal contribution of IMTG to energy supply [7, 18]. This temporal association suggests that lactate accumulation may modulate adrenaline-driven lipolysis in skeletal muscle, though the exact crosstalk between systemic catecholamine release and local lactate signaling requires further investigation. At this point, the role of lactate binding to GPR81 in inhibiting fat breakdown may be less than the role of adrenaline binding to β-AR in promoting fat breakdown, overall promoting fat breakdown. However, In animal studies, based on comprehensive evidence from receptor expression, functional responses, molecular mechanisms, and pathology, the overall intensity of adrenergic signaling in skeletal muscle is lower than that in adipose tissue [32, 82–84]. Moreover, adrenergic signaling in skeletal muscle is more closely associated with glycogenolysis and protein metabolism, rather than directly regulating lipolysis [85–87]. when the blood lactate concentration is greater than 5 mM, the inhibitory effect of the lactate/GPR81 signaling axis on fat breakdown may dominate, overall inhibiting the breakdown of fatty acids. This could also explain the fatty acid contribution of skeletal muscle in Fig. 5, which continues to rise with exercise intensity when the blood lactate concentration is less than 5 mM, and begins to gradually decline after exceeding 5 mM. Specifically, at rest, IMTG in skeletal muscle hardly participates in fatty acid metabolism, but under the stimulation of adrenaline, IMTG begins to gradually break down to supply energy; until the blood lactate concentration exceeds 5 mM, although adrenaline continues to promote the breakdown of IMTG, due to the increase of lactate concentration, its inhibitory effect ultimately limits further breakdown of fatty acids, causing the contribution of fatty acids from skeletal muscle IMTG to gradually decrease.

Now, we believe that the term “mitochondrial function” is somewhat vague and does not accurately describe the specific expressions of mitochondria [23].Therefore, we would like to emphaspertains to mitochondrial energy metabolism and mitochondrial biogenesis, and does not involve cell-dependent properties or molecular features of mitochondria. Created with BioRender.com.

Our hypothesis that endogenous lactate enhances sympathetic activity during exercise remains speculative. Romijn et al.’s data challenge the simplistic “concentration threshold” model [25]. But their data are also favorable to some aspects of our hypothesis. Although systemic glycerol Ra did not differ, at 85% VO_2_max, peripheral (likely skeletal muscle) glycerol Ra increased, whereas adipose tissue glycerol Ra decreased non-significantly. This aligns with our hypothesis that lactate may differentially regulate lipolysis in adipose tissue compared to skeletal muscle. Currently, this hypothesis still faces many challenges. While rodent studies suggest lactate-sensitive central pathways, human data are conflicting, and no study has directly measured adrenaline secretion in response to exercise-induced lactate fluctuations while controlling for confounding factors (e.g., hypoglycemia, baroreflexes). Mechanistic studies using lactate clamp techniques, tissue-specific GPR81 knockout models, and simultaneous measurements of adipose/muscle lipolysis are urgently needed to resolve these complexities.

## Lactate is Involved in Regulating the Mechanism by which Long-term Exercise Promotes the Breakdown of Fatty Acids

**Fig. 6:**
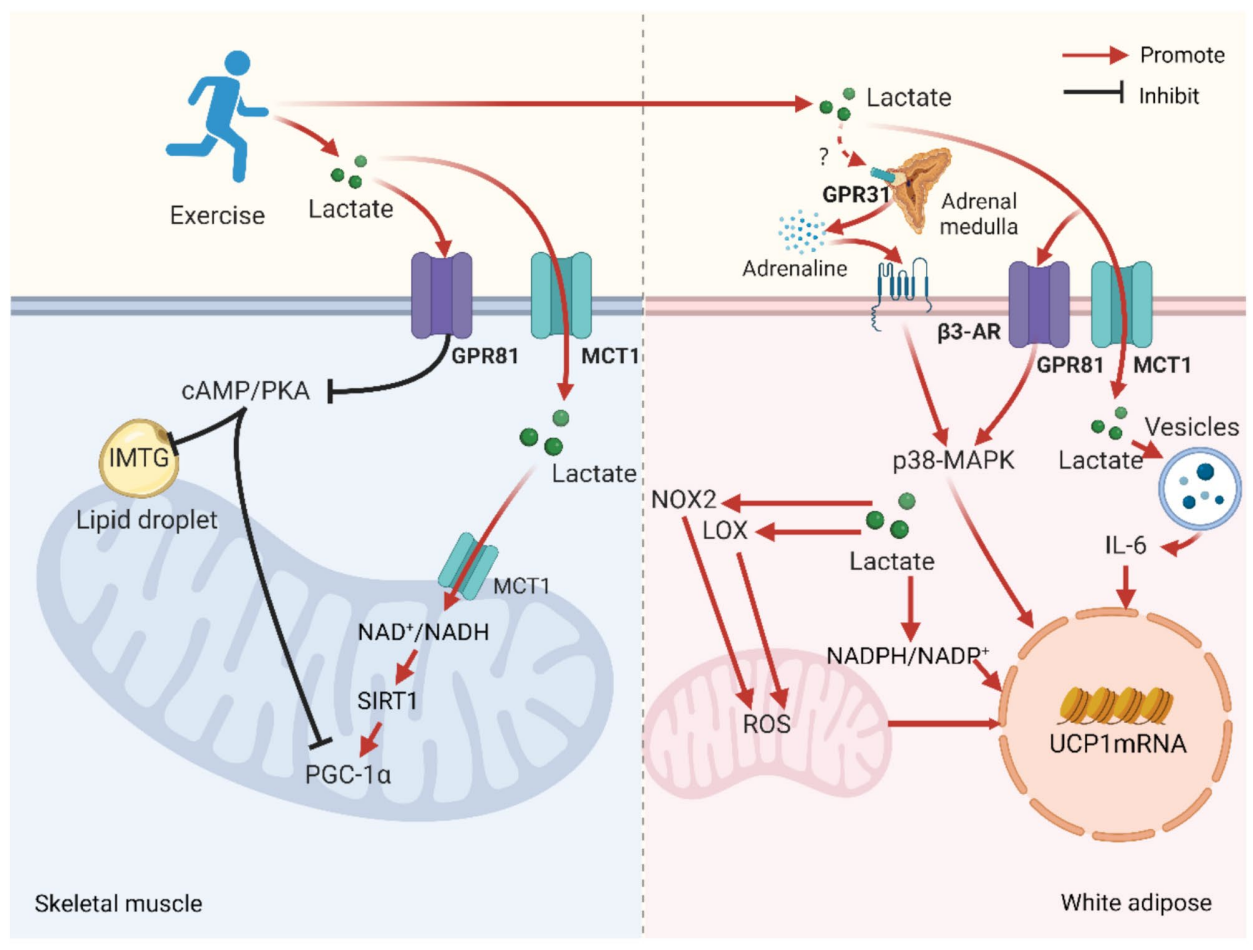
Lactate is involved in regulating the possible mechanisms of long-term exercise-induced fat metabolism in white adipose and skeletal muscle tissue

Studies have shown that mice administered with low concentrations of lactate through injection or intragastric gavage exhibit enhanced breakdown of white adipose tissue, suppressed fat synthesis, and enhanced mitochondrial function [30, 31]. It is well recognized that regular exercise promotes fat breakdown and browning, as well as enhances mitochondrial function. As one of the metabolic products of exercise, does lactate’s signaling functions participate in the regulation of long-term exercise-induced fat breakdown and browning processes? Research has confirmed that lactate is involved in exercise-mediated white adipose tissue browning [11]. Other studies have also found that lactate can promote the browning of white adipose tissue, and the mechanism may be that lactate acts through metabolic pathways (mediated by MCT1) and receptor pathways (GPR81 signaling axis) [43, 88] (see Fig. 6 for details).

Red indicates overall promotion, and blue indicates overall inhibition. During exercise, skeletal muscle is the primary site of lactate production. However, the impact of lactate on skeletal muscle metabolism is multifaceted, involving both direct intracellular utilization and indirect effects mediated by the bloodstream. While lactate is directly utilized within muscle cells, it also diffuses through the cell membrane into the bloodstream, contributing to blood lactate levels. Notably, changes in blood lactate levels can influence the metabolic state of skeletal muscle. For instance, when blood lactate levels rise, skeletal muscle shifts from lactate release to lactate uptake. GPR81 (G Protein-Coupled Receptor 81), MCT1 (Monocarboxylate Transporter 1), cAMP/PKA (Cyclic AMP/Protein Kinase A), IMTG (Intramuscular Triglyceride), NADH (Nicotinamide Adenine Dinucleotide), SIRT1 (Silent Information Regulator Sirtuin 1), PGC-1α (Peroxisome Proliferator-Activated Receptor Gamma Coactivator 1 Alpha), GPR31 (G Protein-Coupled Receptor 31), β3-AR (Beta-3 Adrenergic Receptor), p38-MAPK (p38 Mitogen-Activated Protein Kinase), NOX2 (NADPH Oxidase 2), LOX (Lipoxygenase), ROS (Reactive Oxygen Species), IL-6 (Interleukin-6), UCP1 (Uncoupling Protein 1). Created with BioRender.com.

In white adipose tissue, lactate primarily regulates the breakdown and browning processes induced by long-term exercise. During regular oxidative metabolism, low concentrations of lactate transported into cells via MCT1 can act as a signaling molecule that stimulates vesicular release of IL-6, promoting IL-6 expression, thereby enhancing the transcriptional level of UCP1 [89]. Concurrently, lactate entering the cells can also generate ROS through LOX located in the mitochondrial intermembrane space [90, 91] and activate NOX2 to produce ROS [92, 93], which enhances the activity of UCP1 through sulfenylation [94], inducing the browning process of white adipose tissue [95]. Additionally, lactate can promote the expression of NRF2 through metabolic pathways, thereby inducing the upregulation of UCP1-related genes and promoting the browning of white adipose tissue [66]. Furthermore, β3-AR, which is essential for maintaining fat breakdown and thermogenesis in the human body [11, 96], may also be enhanced by lactate induced by long-term exercise, thus promoting the breakdown of white adipose tissue and thermogenesis of brown adipose tissue [97]. Notably, studies have shown that the GPR81 receptor is significantly reduced in the white adipose tissue of obese mice, and the absence of the GPR81 receptor significantly affects the browning process of white adipose tissue and the thermogenic capacity of brown adipose tissue. However, the use of a β3-AR agonist can significantly increase the expression level of GPR81 in obese mice [98]. This finding highlights the potential role of GPR81 in promoting energy expenditure and adipose tissue metabolism, suggesting a positive regulatory relationship between β3-AR and GPR81. The activation of β3-AR may promote the browning and thermogenesis of adipose tissue by upregulating the expression of GPR81. In the same study, researchers also explored the effect of oral lactate on the browning of white adipose tissue. The results indicated that in this process, p38-MAPK may act as a key downstream signaling molecule common to GPR81 and β3-AR [98]. The activation of p38-MAPK is crucial for the browning process of white adipose tissue, and lactate may affect the activation of p38-MAPK on UCP1 mRNA through both GPR81 and β3-AR pathways during long-term exercise. Therefore, lactate promotes the breakdown and browning of white adipose tissue through both receptor pathways and metabolic pathways.

In skeletal muscle tissue, lactate primarily regulates the accumulation of IMTG and the enhancement of mitochondrial biogenesis and antioxidant function induced by long-term exercise. Lactate inhibits the cAMP/PKA signaling pathway through the GPR81 receptor, leading to the accumulation of IMTG [12, 13, 30, 31]. Concurrently, according to experimental results, the increase in PGC-1α and citrate synthase indicates the enhancement of mitochondrial biogenesis and antioxidant function, which contradicts the hypothesis that lactate binding to the GPR81 receptor essentially inhibits mitochondrial biogenesis in skeletal muscle [30, 31, 99]. This implies that lactate can affect mitochondrial biogenesis through other pathways, promoting mitochondrial function [64]. Studies have pointed out that during exercise, the function of lactate as a fuel substrate and a precursor for gluconeogenesis is enhanced, participating in glycolysis and the TCA cycle as the body’s primary energy substance, increasing the NAD+/NADH ratio, and simultaneously promoting the activity of SIRT1 and the expression of PGC-1α, which promotes mitochondrial biogenesis [100], thereby showing enhanced mitochondrial function. Additionally, research suggests that the cAMP/PKA-dependent activation of p38-MAPK is an indispensable step for the transcription of the UCP1 gene in mice [101]. Exercise enhances the activity of the GPR81 receptor in skeletal muscle without significantly affecting the β3-AR signaling pathway; lactate may inhibit p38-MAPK-induced white fat browning through the GPR81/cAMP/PKA pathway. Therefore, lactate inhibits the breakdown and browning of white fat and mitochondrial biogenesis in skeletal muscle tissue through receptor pathways but promotes mitochondrial biogenesis through metabolic pathways.

In summary, unlike the effects of lactate concentration changes on fat metabolism in white adipose and skeletal muscle tissues during a single bout of exercise, the intake of low concentrations of lactate during long-term exercise exerts multifaceted effects on white adipose and skeletal muscle tissues through both receptor pathways and metabolic routes. It not only promotes the breakdown and browning of white adipose tissue and inhibits fat breakdown in skeletal muscle tissue through these pathways but may also regulate mitochondrial biogenesis through metabolic routes. Although these roles of lactate have been recognized to some extent, the specific mechanisms of action of lactate during long-term exercise and the differential effects in various tissues remain an area that requires further investigation.

## Study Limitations

The current understanding of lactate’s regulatory role in fatty acid metabolism is constrained by several methodological and conceptual limitations that warrant careful consideration. Substantial heterogeneity exists across studies regarding experimental models (ranging from rodent specimens to human athletes), lactate concentration ranges (2–20 mM), and exercise intervention durations (acute bouts vs. chronic training). The field urgently requires standardized experimental frameworks specifying species-specific exercise intensities, tissue sampling timelines, and analytical techniques.

1. While emerging evidence suggests distinct signaling roles for mitochondrial versus cytoplasmic lactate pools, the field lacks direct mechanistic comparisons;
2. the prevailing research focus on canonical pathways (e.g., GPR81-mediated lipolysis inhibition) may overlook alternative regulatory mechanisms. Future investigations employing multi-omics approaches could reveal novel regulatory axes;
3. Our hypothesis regarding the sympathoexcitatory effects of lactate remains empirically untested. While optogenetic studies in rodents have identified lactate-sensitive hypothalamic circuits, human trials have shown conflicting responses. A well-designed cross-sectional study measuring real-time catecholamine release through microdialysis under lactate conditions might be able to resolve this controversy.

## Conclusion

Lactate’s role in energy metabolism during exercise is far more complex than the traditional view of it being a simple metabolic byproduct. Instead, it acts as a key regulatory molecule that finely tunes fatty acid metabolism in white adipose and skeletal muscle tissues, exerting a profound influence on exercise adaptation and metabolic health. Through multiple pathways such as the GPR81 receptor and MCT1, lactate regulates the cAMP/PKA signaling pathway, adrenaline concentration, and mitochondrial function, demonstrating its complex mechanisms of action on fatty acid metabolism during both acute and chronic exercise. These findings emphasize the importance of lactate concentration dependence and exercise duration dependence in the regulation of fatty acid metabolism, indicating significant differences in the effects of lactate on white adipose and skeletal muscle tissues at different exercise intensities. At low to moderate exercise intensities, lactate promotes the release and utilization of fatty acids, while at high-intensity exercise near or exceeding the lactate concentration threshold, its role shifts to inhibiting fatty acid breakdown. This shift is crucial for understanding exercise adaptation and the development of exercise training programs.

Moreover, this review summarizes the key role of lactate in the long-term exercise-induced restructuring of fatty acid metabolism, particularly in promoting the browning of white adipose tissue and enhancing mitochondrial biogenesis and antioxidant function. This provides new perspectives and potential targets for the prevention and treatment of obesity and metabolic syndrome. Given the multifaceted and complex nature of lactate in energy metabolism, future research should further explore the metabolic regulatory mechanisms of lactate under various physiological and pathological conditions, as well as how it interacts with other metabolic signaling molecules to collectively affect energy balance and metabolic health. This will not only enrich the understanding of exercise metabolism regulation but also provide a scientific basis for the development of new exercise training methods and treatment strategies for metabolic diseases.

## Data Availability

Not applicable.

## Abbreviations

β3-AR: Beta-3 adrenergic receptor
ACC1: Acetyl CoA carboxylase
AMPK: Adenosine 5’-monophosphate (AMP)-activated protein kinase
ATGL: Adipose triglyceride lipase
ChREBP: Carbohydrate response element binding protein
CPT1: Carnitine Palmitoyl Transferase 1
ETC: Electron Transfer Chain
FAT/CD36: Fatty acid translocase
FFA: Free fatty acids
G3P: Glyceraldehyde 3-phosphate
GPD1: Glycerol-3-phosphate dehydrogenase 1
GPR81: G protein-coupled receptor 81
HSL: Hormone-sensitive triglyceride lipase
IMTG: Intramuscular triglycerides
LOX: Lactate oxidase
MG: Muscle glycogen
MRS2: Mitochondrial RNA splicing 2
mTORC2: Mammalian target of rapamycin complex 2
NADH: Nicotinamide Adenine Dinucleotide
NADPH: Reduced nicotinamide adenine dinucleotide phosphate
NOX2: NADPH Oxidase 2
NRF2: Nuclear factor erythroid 2-related factor 2
OXPHOS: Oxidative Phosphorylation
p38-MAPK: p38 mitogen-activated protein kinase
p70S6K: p70 Ribosomal protein S6 kinase
PGC-1α: Peroxisome proliferators-activated receptor γ coactivator 1α
Plasma FFA: Plasma free fatty acids
Plasma G: Plasma glucose
SCD1: Stearoyl-CoA desaturase 1
SIRT1: Silent information regulator 1
SREBP-1c: Sterol regulatory element binding protein-1c
TG: Triglyceride
UCP1: Uncoupling protein 1
